# Direct Retrieval of Biomechanical and Hydrodynamic Parameters for Drug Carrier Liposomes Using Conventional Extrusion Processes

**DOI:** 10.1021/acsomega.5c11079

**Published:** 2026-01-20

**Authors:** Maria Victoria Heiderick Machado, Maria Luiza Barbosa Pertence, Caroline Mari Ramos Oda, Jaqueline Aparecida Duarte, Ubirajara Agero, Elaine Amaral Leite, Angelo Malachias

**Affiliations:** † Departamento de Física, ICEx, 28114 Universidade Federal de Minas Gerais - UFMG, Av. Antonio Carlos, 6627, Belo Horizonte - MG, CEP 30123-970, Brazil; ‡ Faculdade de Farmácia, Universidade Federal de Minas Gerais - UFMG, Av. Antonio Carlos, 6627, Belo Horizonte - MG, CEP 30123-970, Brazil

## Abstract

Physical parameters such as membrane elasticity and solution viscosity in a liquid medium play crucial roles in the effectiveness of drug delivery. Liposome formulations, used in both research and clinical contexts, are usually designed to achieve desired chemical stability, particle size, and drug encapsulation efficiency. However, meeting such requirements may not suffice in order to succeed in in vivo tests, which can be frustrated due to poor evaluation of biomechanical conditions. In this work, we introduce simple biomechanical evaluation protocols that make use of conventional pressure-based liposome extrusion as well as dynamic light scattering results to extract elastic (mechanical) and hydrodynamic (viscosity) properties of colloidal solutions of liposomes. We describe a sequence of analytical steps that need to be carried out in order to obtain macroscopic results that are directly comparable to those of other methods. Two distinct and complementary procedures are presented: the first uses a systematic variation of extrusion pressure, giving access to the viscosity of the solution, and the second being a statistical evaluation of the particle size distribution obtained by dynamic light scattering, providing elasticity constants for liposomal systems. Both methods carry the advantage of generating results for the liposome suspension that will be applied to real systems, thereby offering a more realistic and integrative characterization compared with microscopic techniques that usually present incomplete statistical coverage.

## Graphical abstract

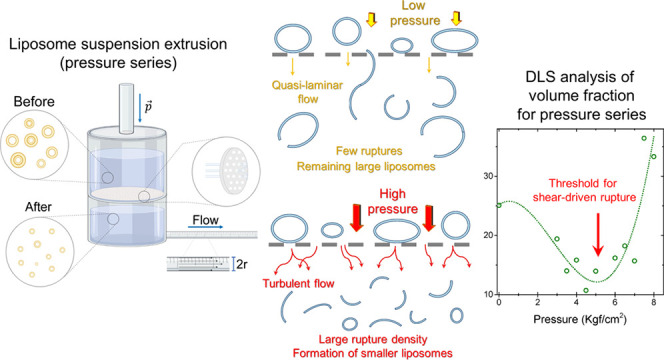

## I Introduction

Liposomes are spherical structures composed of one or more organized lipid bilayers (of amphiphilic molecules) enclosing an aqueous internal cavity. Such amphiphilic structure, combined with their biocompatibility, makes liposomes highly promising drug delivery systems for water-soluble compounds (usually stored inside their cavity) as well as water-insoluble chemicals (which can be stored between lipid layers).^1^ A broad spectrum of therapeutic and diagnostic agents can be effectively delivered by using liposomal systems. These include antibiotics,^2^ proteins,^3^ peptides,^4^ dyes,^5^ nucleic acids,^6^ antioxidants,^7^ enzymes,^8^ antimicrobials,^9^ antifungals,^10^ antivirals,^11^ antitumor drugs,^12^ gene delivery,^13^ immunotherapy compounds,^14^ antioxidants,^15^ antiaging agents,^16^ and even mRNA vaccines.^17^

Although other types of nanometer-sized carriers have been recently studied in medicine and antitumor therapies,^18–21^ the use of liposomes remains advantageous. Inorganic nanoparticles can carry approximately 5% of their weight^22^ due to surface/volume geometrical limitations. Gold nanoparticles, for instance, usually exhibit relevant issues concerning toxicity and biodistribution.^23,24^ Considering organic self-assembled objects, the use of micelles as drug carriers also presents inherent stability problems, such as shell rupture before reaching the target tissue.^25^

Focusing on the use of liposomes in antitumor therapies, which are usually limited due to drug toxicity, they exhibit unmatched versatility. Liposomes can carry a plethora of molecule types,^26,27^ are easily functionalized with respect to their surface properties, and have chemical potential tunability to reach target tissues.^28,29^ Liposomes can perform controlled release of drugs over an extended time lapse^30–32^ and have inherent low cytotoxicity due to flexible biocompatibility parameters.^33^ Finally, liposomes can be used for both passive and active targeting, using the effect of enhanced permeability and retention of tumors (in the first case) as well as surface functionalization (in the second scenario).^34^

The ability to release drugs in specific target tissues is a crucial property of liposomal formulations, achieved as a successful combination of the desired surface chemical potential and viscoelastic properties. In particular, viscoelastic properties are often tailored after unsatisfactory in vitro or in vivo tests, being considered in a later thorough investigation of biochemical parameters. The purpose of the present work is to provide a reliable and simplified protocol to evaluate the viscoelastic properties of liposomes in solution. The retrieved information adds degrees of tunability that go beyond the usual zeta potential, polydispersity index, and average liposome size evaluation. Our suggested methodology is based on a stepwise pressure extrusion series with simultaneous and direct viscosity flow assessment, followed by additional (unconventional) statistical analysis of the dynamic light scattering size distribution. Using these simple procedures, available in most laboratory facilities dedicated to liposome drug delivery investigation, one can determine viscoelastic parameters that are crucial for drug encapsulation and delivery efficiency.

## II Theoretical Background: Viscosity and Elasticity

### II.a Fluid Flow: Poiseuille’s Law

Poiseuille’s law outlines one of the simplest procedures to measure the viscosity of a fluid. It describes the laminar flow of an incompressible fluid subjected to a hydrostatic pressure *P* that results in a volumetric flow rate *Q*. Considering a Newtonian fluid in a pipe with radius *r* and length *L*, with a total fluid volume *V* and a flow rate *t* (*Q* = *V*/*t*), the dynamic viscosity η can be calculated from^35^

1 $$\eta =\frac{\pi tPr^{4}}{8VL}$$

Other terms can be added to Poiseuille’s law in order to take into account non-Newtonian fluids. Such additional terms depend on the fluid behavior, modifying eq 1 for particular cases. For a fluid in which viscosity follows a power law equation, the resulting equation for the flow rate is written as

2 $$Q=\frac{\pi tr^{3}}{\left(3n+1\right)}{\left(\frac{rP}{2KL}\right)}^{1/n}$$

where *Q* is the volumetric flow rate, *K* is the apparent viscosity of the fluid (expressing the η dependence on shear), and *n* is an integer number that depends on specific properties of the non-Newtonian fluid.^36^ In this work, we use an extrusion setup where the fluid is subjected to controlled pressure and flows through a pipeline with well-defined dimensions. By monitoring the fluid flow speed, one can extract its viscosity. Although the models represented in eqs 1 and 2 are approximations, relative variations of the flow rate from distinct fluids may be ascribed to changes in physical parameters for liposomal formulations under practical conditions. In this work, the liposome suspensions evaluated are >99% saline solution (by volume), presenting Newtonian fluid behavior. Small deviations from a Newtonian fluid do not modify our conclusions, but additional considerations may be required for much less hydrated sample conditions.

### II.b Continuum Elasticity and Young Modulus

In order to extract elastic properties of lipidic membranes, one must compare them quantitatively with other known materials. Such a comparison is crucial since strain–stress measurements on biological systems usually present large deviations due to fluctuations of sample composition as well as methodological indeterminations due to large size distributions or limited sampling of analyzed systems. Here we use a semiquantitative approach to directly access the shear strain γ^37^ and infer Young modulus *E*.^38^ The Young modulus is the major parameter of elasticity in any system since it relates the strain of a material induced by the stress application along the same axis (or direction), providing its mechanical resistance. For inorganic materials, the Young modulus is usually anisotropic (depending on atomic crystalline organization) and ranges from a few tens to hundreds of GPa. In biological systems and organic materials, usually values in the range of MPa are retrieved.^39^

Although a direct measurement of the Young modulus in liposomes may require complex measurement systems, the shear strain γ (as well as the flexure modulus *k*_c_) can be estimated using the strength (shear threshold) observed in consolidated processes using tools such as optical tweezers.^40^ Power-law relations of *E* and γ show universal behavior for both inorganic and biological compounds.^41^ A scaling factor of nearly 100 is found between both variables: a material with a value of *E* = 10 MPa usually yields γ ≈ 0.1 MPa. A similar proportion (30 to 100 times) is also found between the Young modulus and flexural strength (also usually referred to as the flexure modulus, *k*_c_) in organic materials.^41^ Although this relation may imply imprecision concerning the absolute value of *E* for a particular system, the relative variation of these constants can be determined if distinct materials are compared. The possibility of extracting a relative factor is crucial to understanding whether the rupture stress threshold of a liposome used for drug delivery is suitable for a given application.

A quantitative comparison with other techniques may lie in the conversion of the flexure modulus *k*_c_ to *E*. The relation between both is provided for membranes by^38^

3 $$k_{c}=\frac{Eh^{3}}{12\left(1-\nu^{2}\right)}$$

where *h* is the membrane thickness and ν is the Poisson ratio for the studied material (usually ν = 0.5 for a membrane in a liquid medium, free to deform transversally upon longitudinal stress).

### II.c Defocusing Microscopy

Defocusing microscopy (DM) is a technique based on retrieving images of phase objects under controlled defocused conditions. In this configuration, the incident light undergoes a phase shift as it propagates through the studied objects, generating contrast, while the total light intensity remains unaffected. The contrast obtained in DM is mathematically proportional to the Laplacian of the phase difference induced by the studied object.^42^ This contrast is usually captured for organic structures by high-speed CCD (200 Hz frame rate) as a grayscale figure.

The formalism used in our analysis was first developed for human red blood cells, for which the membrane resistance to thermal fluctuations due to Brownian motion in a liquid environment is associated with local curvature energy, as proposed by Brochard and Lennon in 1975.^43^ They proposed a bending fluctuation spectrum (*u*(*q*)) model expressed by

4 $$\left|{u\left(q\right)}^{2}\right|=\frac{k_{B}T}{k_{c}q^{4}}$$

where *k*_c_ is the bending (curvature) modulus), *k*_B_ is the Boltzmann constant, *T* is the temperature, and *q* is the wavevector associated with the observed membrane fluctuation.

The contrast time-dependent correlation of the object membrane at a time *t* and its condition at *t* = 0 depends exponentially on the bending modulus *k*_c_ and the membrane viscosity η, resulting in the relation

5 $$\langle \Delta C\left(0,0\right)\Delta C\left(0,t\right)\rangle ={\left(\frac{\Delta n}{n_{0}}\right)}^{2}\left(\frac{{z_{f}}^{2}}{4\pi }\right)\left(\frac{k_{b}T}{k_{c}}\right)\int_{0}^{q_{máx}}qe^{-k_{c}q^{3}t/4\eta }dq$$

where Δ*n* is the difference between the index of refraction of the phase object and the liquid environment, *q* is the fluctuation wavevector, *k*_0_ is the light incident wavevector (in air), and *z*_f_ is the defocusing with respect to the object focal plane of minimum contrast. The deduction of eq 5 can be found in ref 44.

## III Experimental Section

### III.a Liposome Synthesis

Blank liposomes of EPC (egg phosphatidylcholine), DPPC (dipalmitoylphosphatidylcholine), and EPC:CHOL (egg phosphatidylcholine and cholesterol, molar ratio 6:4) were prepared by the lipid film hydration method.^45^ The total lipid concentration defined for each formulation was 10 mM for all of the formulations. CHOL, EPC, and DPPC were dissolved in chloroform and transferred to a round-bottom flask. The solvent was removed under reduced pressure using a Buchi Labortechnik AG Rotator CH-9233, model R-210, coupled to a V-700 vacuum pump (Flawil, Switzerland) for 2 h. In our synthesis, the rotary evaporator was used at a rotating speed of 130 rpm while being kept at 30 °C under a partial pressure of 131 mbar. After complete solution evaporation, the thin films of liposomes were hydrated using a 0.9% (w/v) NaCl solution under agitation. The proportional volume of chloroform and saline solution was 0.3 mL of chloroform for each 1.0 mL of NaCl (0.9%) solution.

The composition of each formulation was chosen based on the studies of Fujisawa et al., Andrade et al., and Rossi et al.^46–48^ Liposome zeta potentials (ZP) for each formulation were measured with a Nano ZS 90 Zetasizer (Malvern Instruments, England), yielding the following values: (i) ZP_DPPC_ = −5.6 mV; (ii) ZP_EPC_ = −2.8 mV; and (iii) ZP_EPC:CHOL_ = −3.9 mV.

### III.b Extrusion Setup and Outlet Fluid Speed

Liposome formulations were extruded with analytical nitrogen using a Lipex Biomembrane extruder, model T001 (Vancouver, Canada). We used an analytical-grade nitrogen cylinder for the extrusion procedures. The extrusion pressure is obtained from a gas pressure regulator with resolution of 0.25 kgf/cm^2^. Prior to the application of each extrusion pressure, the nitrogen is released from the pipeline and a new pressure is set until it reaches a full port ball valve located at the inlet port of the extruder vessel. The valve is then opened, allowing the pressure to enter the extruder. Since the output pipeline had a reduced gauge (diameter), no drop from the applied pressure was observed along the whole process until the suspension content was fully extruded into a beaker.

For each liposome suspension studied in this work, the extrusion pressure was systematically varied in the range from 2.0 to 12.0 kgf/cm^2^ with pressure steps of 0.5 kgf/cm^2^ (twice the resolution of 0.25 kgf/cm^2^). Liposome formulations were extruded through 0.2 μm (200 nm) polycarbonate membranes and replaced by new membranes after each extrusion, and the volume of single-pass extruded solution was reserved for dynamic light scattering analysis.

### III.c Dynamic Light Scattering

The mean diameter and the polydispersity index (PDI) of liposomes before and after extrusion were determined by dynamic light scattering (DLS), at 25 °C, at an angle of 90°. DLS measurements were carried out, in triplicate, in a Nano ZS 90 Zetasizer (Malvern Instruments, England). Dispersions of each liposome type were analyzed for each single-pass extrusion condition (defined as a combination of liposome and extrusion pressure) as well as for the as-synthesized formulations. The DLS evaluation procedure for each suspension was carried out in 11 runs, each with 3 measurements of 10 s accumulation time (therefore a total of 33 measurements). A pre-equilibration time of 120 s was used prior to every run. Solvent viscosity was measured in our work as 1.01(0.10) cP and is comparable to values retrieved by the DLS equipment of 0.98(3) cP.

### III.d Defocusing Microscopy setup

In order to characterize the membrane fluctuations, providing a quantitative measurement of liposome viscosity and curvature modulus, liposomes were filmed at a capture rate of 200 Hz for 30 s. For imaging, liposomes were deposited in 35 mm glass bottom dishes (Cellvis) containing a 20-mm-diameter coverslip at the base. Microscopy measurements were performed in a Nikon Eclipse TI inverted microscope, a stage-heated oil immersion objective Nikon Apo TIRF 100×, NA 1.49 (Nikon, Japan), 25 °C temperature, and 50% humidity. The images were analyzed with the ImageJ software and a MATLAB script based on results from ref 49.

In eq 5, we used *n*_0_ = 1.508 ± 0.001 as the refractive index of the immersion medium and Δ*n* = 0.06 as the difference between the refractive index of the phase object and the surrounding medium. A thermal energy of *k*_B_ *T* = 4 × 10^–21^ J was considered, along with a standardized defocusing distance of *z*_f_ = 1 μm. For our optical microscopy setup, the upper limit of the wavevector (*q*_max_) was determined through computational tests using red blood cells as a reference object by analyzing the point spread function and estimating the diameter of the Airy disk, establishing a practical resolution limit corresponding to *q*_max_ ≈ 4 μm^(−1)^ that accurately reflects the true resolving power of the microscope.^49^

## IV Results

A representation of the liposome extrusion process is given in Figure 1(a). In our system, the gas (analytical nitrogen) inlet has a variable pressure that can be fixed at any value in the range from 2 to 12 kgf/cm^2^, with divisions of 0.25 kgf/cm^2^. The extruded dispersion outlet is attached to a silicone tube with an internal diameter of 0.15 and a 30 cm length.

**Figure 1:**
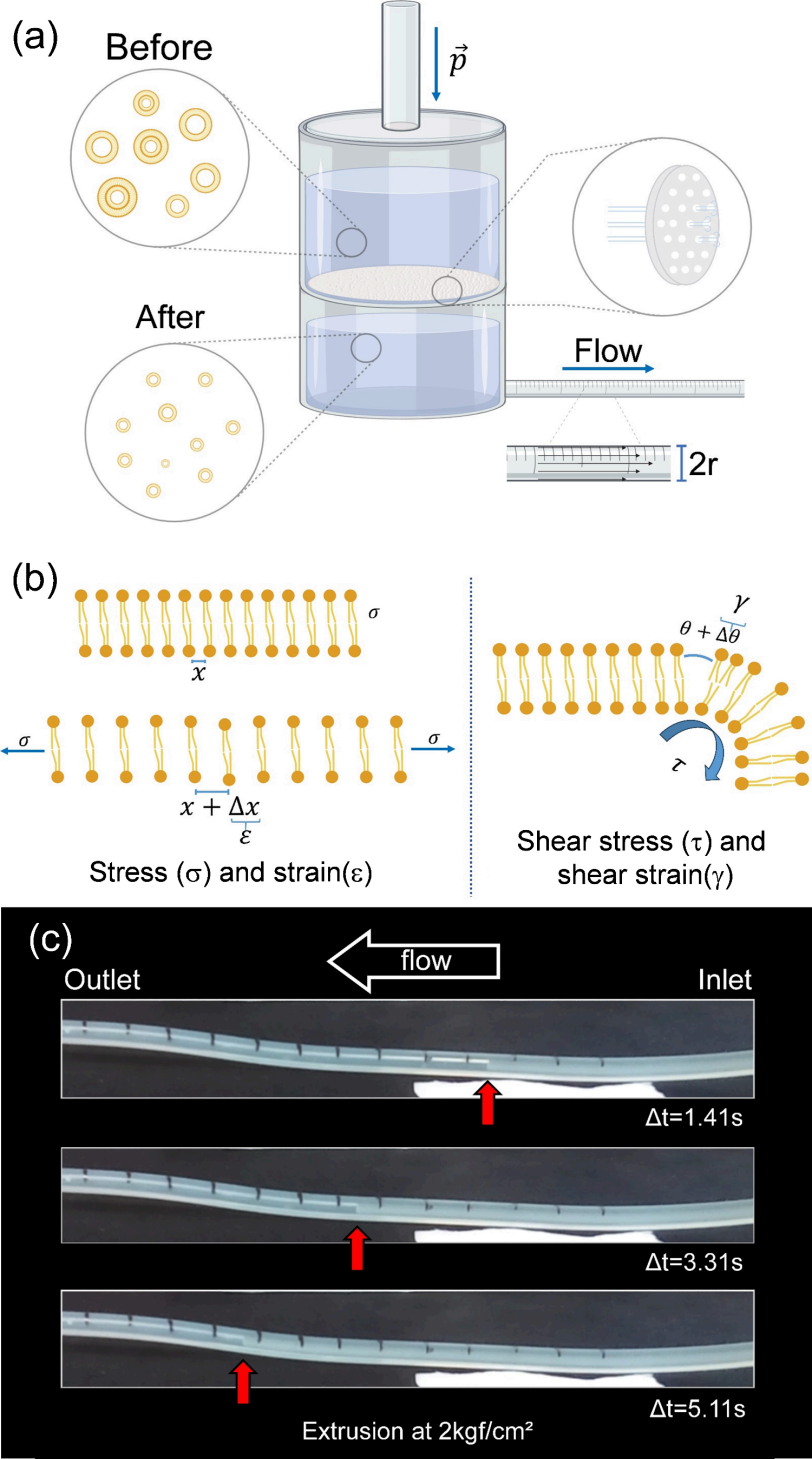
(a) Schematic representation of the extrusion setup in which a fixed N_2_ pressure is used to pull a liposome solution through a 200 nm pore size membrane. In these processes, the extruded liposomes are mechanically forced to a size reduction and the solution flows through a silicone pipe with marks for each centimeter length. (b) Representation of deformation possibilities for lipid membranes that lead to membrane rupture upon extrusion. In-axis stress (σ) and strain (ε) are represented in the left panel, while shear stress (τ) and shear strain (γ) are represented in the right panel. (c) Formulation flow through the extruder output pipe captured with a 120 frame per second camera. The tube inlet and outlet are located on the right and left sides of the snapshot panels, respectively. The position of the solution meniscus is marked with red arrows for different times at a fixed extrusion pressure of 2 kgf/cm^2^ for the EPC liposome suspension.

After passing through a 200 nm pore-size membrane, the liposomes contained in the extruded colloidal suspension have a reduced diameter due to a shear process that takes place in their membrane pores. The microscopic strain–stress and shear phenomena are represented in Figure 1(b). Although both normal strain and shear strain can take place along extrusion procedures, the dominant mechanism for membrane structural rupture and reorganization is the shear strain γ.^37^

The flow in the postextrusion tube can be monitored using a camera with a capability of up to 120 frames per second (camera time resolution better than 0.01 s). Since the flow speed depends on the viscosity of the suspension, one can evaluate it quantitatively by monitoring the fluid passage through centimeter-spaced marks drawn along the tube, as shown in Figure 1(c). In this figure, selected snapshots of the EPC:CHOL formulation extrusion are shown for a fixed pressure of 2 kgf/cm^2^. We have chosen to illustrate the extrusion process of the EPC:CHOL formulation, considering *t* = 0 s to be the exact moment when the solution leaves the extruder and starts to flow in the output opening of the pipeline. Extrusion times depicted in Figure 3(c) of 1.41 3.31, and 5.11 s are illustrative of the flow rate for this fixed pressure, indicating the movement of the liquid front meniscus, used to calculate the suspension viscosity (time values are evaluated after the liquid leaves the output opening of the extruder). Several short footages were carried out, accounting for each of the distinct pressure values in each liposome formulation. The camera was positioned to capture the extrusion outlet port and the overall tube length.

The analysis of the flow time for each of the liposome suspensions and their comparison with the bare saline medium (without liposomes) is used for viscosity analysis. For EPC and EPC:CHOL, the usual time for liquid flow along a 15 cm path of a 1.50(5)-mm-diameter tube was found to be 1 to 2 s for a pressure of 4 kgf/cm^2^. In the DPPC suspension, a flow time of a few minutes was observed (which already indicates the larger values for viscosity in this system). In all systems, one observes that for large pressure values, considering a fixed extrusion time, the suspension travels a larger distance through the output tube (larger pressures produce faster flow speed).

Using this simple video capture procedure, the average suspension flow speed is calculated by considering the time required for the suspension to pass 15 marks distanced from 1 cm each. The result for the 0.9% NaCl solution (without liposomes) is shown in the graph in Figure 2(a). The data set exhibits fairly linear behavior, and a linear fit yields a slope of 6.9(8) cm·kgf^–1^·s^–1^. The obtained slope directly relates to the viscosity using Poiseuille’s law of eq 1

6 $$A_{slope}=\frac{\pi r^{4}}{8V\eta }$$

where *V* is the total liquid volume that goes through the tube length, *r* is the outlet tube radius, and η is the dynamic viscosity. Therefore, in such a representation, large slope numbers correspond to small dynamic viscosity values. Using this procedure, one obtains η_saline_ = 1.01(0.10)­cP.^50^ The value retrieved cannot be interpreted on an absolute scale but serves as a reference for the calculation of the relative viscosity proportion for liposome formulations (which are indeed made out with saline solution).

**Figure 2:**
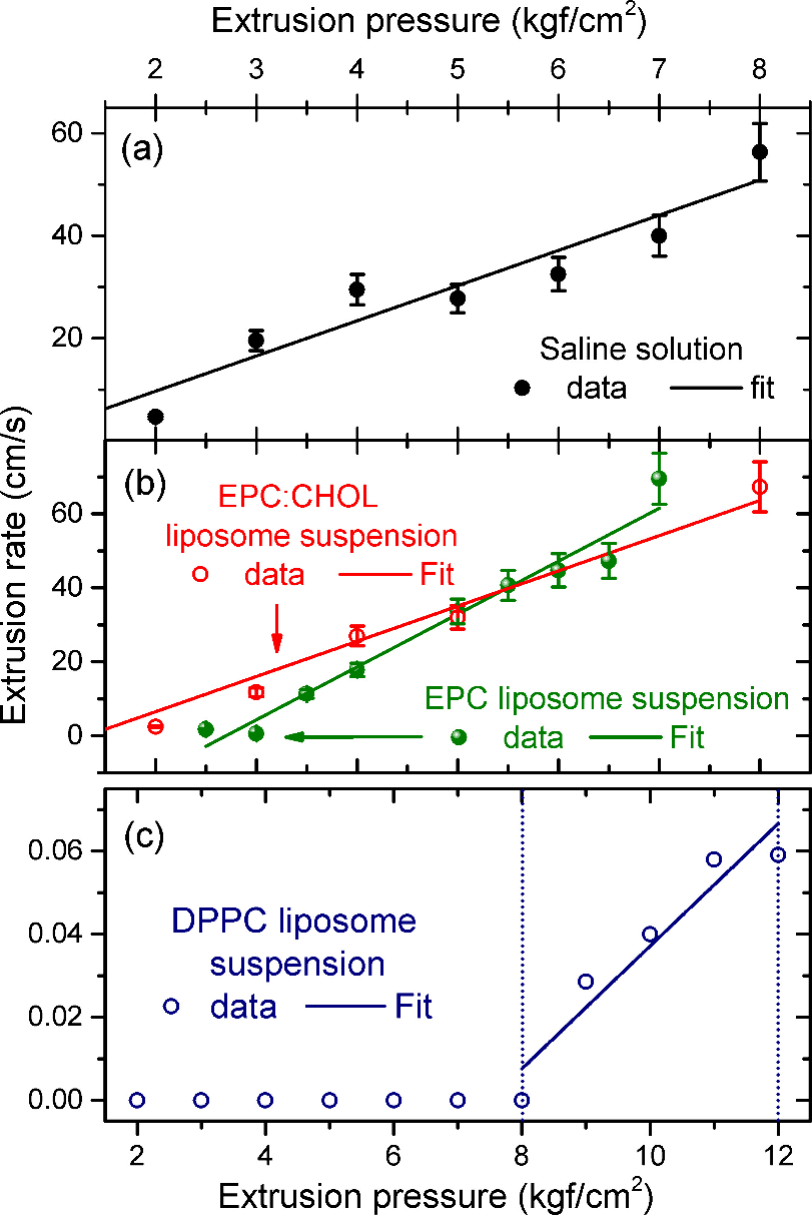
Extrusion rate data for different extrusion pressures ranging from 2 to 8 kgf/cm^2^ (range extended for 12 kgf/cm^2^ in the DPPC suspension) for the liposome systems studied in this work: (a) reference saline solution; (b) EPC and EPC:CHOL liposome; and (c) DPPC liposome (error bars are smaller than symbol sizes in this panel). In all cases, a linear fit was used to extract the viscosity of the solution under conditions where the extrusion rate was nonzero. Error bars are represented in cases where their size is larger than the symbol size in all panels.

Figure 2(b) shows the same flow results and fit for the EPC and EPC:CHOL liposomes. The retrieved slopes from fits are 14.3(1.0) and 9.5(1.3) cm·kgf^–1^·s^–1^, yielding η_EPC_ = 0.49(5)­cP and η_EPC:CHOL_ = 0.96(13)­cP, respectively. These results indicate that both liposomes decrease the viscosity by approximately 48% (EPC) and 4.9% (EPC:CHOL).

Finally, Figure 2(c) shows the extrusion result for DPPC. In this case, the extrusion is feasible only for pressures above 8 kgf/cm^2^. The small slope value retrieved in our experiment, 0.015(3) cm·kgf^–1^·s^–1^, indicates a much larger value of viscosity, which reaches η_DPPC_ = 633(60) cP in our calculations. We believe that this value does not relate to the real viscosity since there is intrinsic difficulty in making the formulation flow through the 200-nm-pore-size membrane. Hence, membrane blocking due to material accumulation is an additional factor that cannot be directly taken into account for our model, restricting its validity range to conditions with viscosity near the saline solution values. It is nevertheless clear that the DPPC viscosity is, in a lower bound estimation, at least 10 times (1 order of magnitude) more viscous than EPC formulations.

Along the extrusion process, the resulting suspensions for each formulation and extrusion pressure are reserved and used in DLS analysis. Usually, extrusion processes are carried out in successive steps, narrowing a large liposome size distribution and reaching conditions with liposome sizes limited to an upper limit near the membrane pore size. A result of DLS size distribution profiles in our EPC:CHOL formulation before and after 10 extrusion steps is shown in Figure 3(a). One observes three maxima at the distribution, located near liposome diameters of 300, 900, and 5500 nm. After successive extrusions at 8 kgf/cm^2^, a single DLS peak is retrieved, centered near the 200 nm liposome diameter.

**Figure 3:**
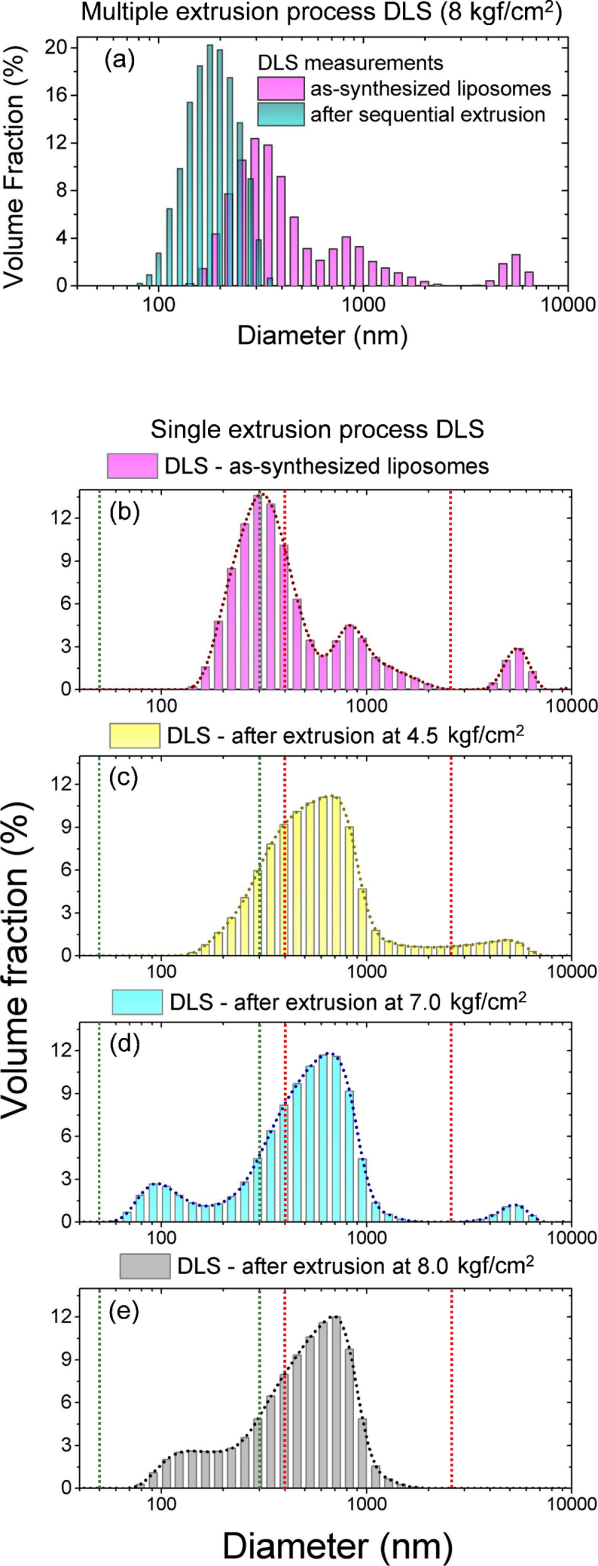
(a) DLS size distribution of the EPC:CHOL liposome formulation before (as-synthesized) and after 10 successive extrusion processes at 8 kgf/cm^2^. Panels (b–e) show DLS results of single-extrusion processes at distinct extrusion pressures for the EPC:CHOL formulation. Size windows for the analysis carried out in Figure 4 are delimited in these panels by the dotted green and red lines (see the text for details). (b) DLS of as-synthesized EPC:CHOL liposomes. (c) DLS of EPC:CHOL liposome solution after a single extrusion at 4.5 kgf/cm^2^. (d) DLS for EPC:CHOL liposome solution after a single extrusion at 7.0 kgf/cm^2^. (e) DLS for EPC:CHOL liposome solution after a single extrusion at 8.0 kgf/cm^2^.

Since we are interested in a deeper understanding of elastic properties, we have carried out single-process extrusion procedures, followed by DLS measurements along the pressure interval. Selected results for the EPC:CHOL liposome formulation are shown in Figure 3(b–e). In these graphs, two size interval windows, ranging from 60 to 300 nm (green dotted vertical lines) and from 400 to 2500 nm (red dotted vertical lines), were set for a pressure-dependent volume fraction analysis. One observes that the liposome distribution of Figure 3(b) (as-synthesized) is modified after a single extrusion at 4.5 kgf/cm^2^, as shown in Figure 3(c). An increase in the area below the graph in the range of 400–2500 nm (large liposomes) is observed. For single extrusions at 7 kgf/cm^2^ [Figure 3(d)] and 8 kgf/cm^2^ [Figure 3(e)], a clear reduction of the area below the DLS distribution is observed for values above 1000 nm, with a consistent increase in the volume fraction of small liposomes in the range from 60–300 nm diameter.

A compound scenario providing the DLS fraction for large EPC:CHOL liposomes and small EPC:CHOL liposomes is depicted in Figure 4(a,b) for all pressures used for extrusion in the range of 2–8 kgf/cm^2^. One notices that for the large liposome windows there is an increase in the volume fraction up to 5.5(5) kgf/cm^2^, followed by a pronounced decrease in this liposome population from 6 to 8 kgf/cm^2^. A reverse (and complementary) trend is observed in the small liposome window, with an increase in the small liposome population for pressures above 6 kgf/cm^2^. A phenomenological interpretation of this observation is provided in Figure 4(c–e). When the polydisperse synthesized liposome formulation is subjected to a single-pass extrusion process (Figure 4(c)], two distinct processes may take place, depending on the pressure and liposome elasticity. For low-pressure values [Figure 4(d)], the formulation flows through the membrane mesh as a quasi-laminar flux, pressing together liposomes that can merge after going through the mesh under the condition of gentle rupture followed by reassembly with neighboring lipidic material. This quasistatic process induces the formation of larger liposomes. At higher pressure values, the flux may become turbulent, increasing the density of ruptures in large liposomes and avoiding the reassembly of their parts in large structures, which in turn favors the formation of small liposomes. A threshold pressure among these conditions is found at the inflection of the volume fraction curves from DLS, which can be interpreted as the upper limit of elastic deformation in the system and related to the strength of the membrane material. The membrane rupture is then expected to take place whenever pressure values are above the shear modulus of the liposomes.^37^ In natural materials and polymers, usual values of shear modulus are found in the range of 1–10 MPa (roughly 1–10 kgf/cm^2^, as observed in our extrusions), while the Young modulus (*E*) for these materials ranges from 0.1 to 1 GPa. Using this approximation, we have established a simplified factor of 100, used to multiply the inflection pressure of DLS volume fraction curves to provide an estimation for absolute values of *E*. It is important to keep in mind here that a precise measurement of *E* (expressed in eq 3) in a liposome suspension usually relies on incomplete methodologies that probe a limited number of structures (such as defocusing microscopy or AFM). Nevertheless, the most important characteristic of the present work is to directly evaluate the relative increase (or decrease) in *E* of a given liposome suspension with respect to a chosen reference system (presently, other liposome suspensions). Under such conditions, where the evaluation of relative values provides sufficient evidence of biomechanical changes, our method shows results comparable to those of well established techniques such as defocusing microscopy (Discussion section). The methodology depicted here has an inherent advantage of using a large (and therefore statistically relevant) volume of a liposome suspension.

**Figure 4:**
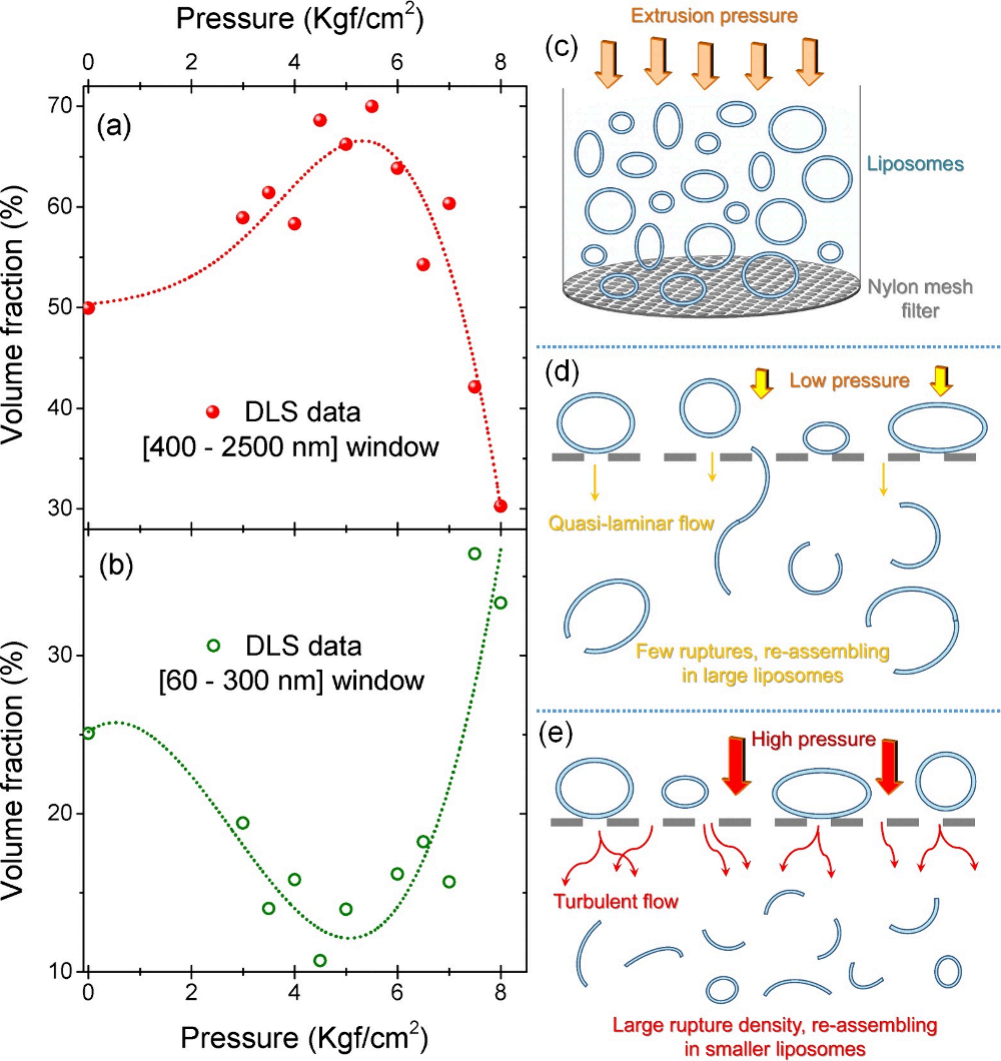
(a) Volume fraction of large EPC:CHOL liposomes evaluated from DLS [red dotted line interval in Figure 3(b–e)] for different extrusion pressures. (b) Volume fraction of small liposomes from DLS data [green dotted line size window in Figure 3(b–e)] as a function of extrusion pressure. (c) Representation of liposome extrusion through a mesh. (d) Possible scenario of reduced rupture density and quasi-laminar flow in reduced extrusion pressures. (e) Suggested extrusion scenario for high pressure, with turbulent flow and large membrane rupture density.

As one changes the liposome composition, using a formulation with reduced or enhanced elastic constants, modifications in the extrusion-pressure series are expected. In fact, it is well known that some liposome formulations are extruded only at high pressures under heating^51,52^ while other formulations are easily extruded at mild pressure values. In our study, these distinct scenarios were probed using the EPC formulation (reduced elastic constants/strength) and DPPC (enhanced elastic constants/strength). The DLS series for a sequence of different extrusion pressures is shown in Figure 5(a, b) for the large liposome window in EPC and DPPC formulations (respectively) and in Figure 5(c, d) for the small liposome window in EPC and DPPC. In both liposome formulations, it is easier to visualize the inflection of the curve by looking at the small liposome window. The condition for rupture is marked by arrows in both cases, yielding values of 3.5(5) kgf/cm^2^ for EPC and 11.0(5) kgf/cm^2^ for DPPC. In the latter, the limitation of the pressure at 12.0 kgf/cm^2^ (due to standard laboratory safety conditions) limited the exploration of higher pressure values, indicating that the Young modulus for DPPC membranes could be related to even larger values.

**Figure 5:**
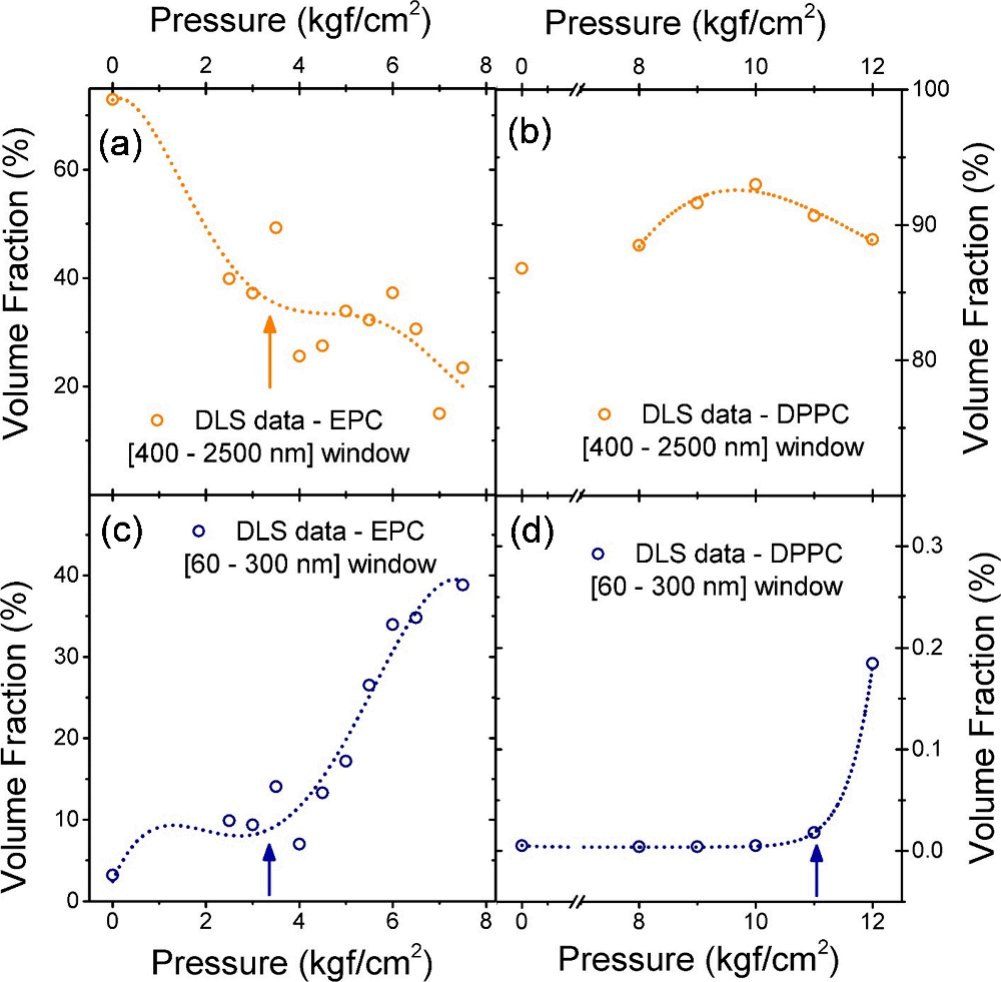
(a) Volume fraction of large liposomes evaluated from DLS for the EPC suspension. (b) Volume fraction of large liposomes evaluated from DLS for the DPPC suspension. (c) Volume fraction of small liposomes evaluated from DLS for the EPC suspension. (d) Volume fraction of small liposomes evaluated from DLS for the DPPC suspension.

## V Discussion

Considering the pressure-dependent extrusion analysis carried out here, the results for dynamic viscosity η, flexure modulus *k*_c_, and Young modulus *E* are listed in Table 1. In all cases, the Young modulus (and consequently *k*_c_) was estimated by multiplying the extrusion threshold pressure by a factor of 100, allowing a rough estimation of values that can, nevertheless, be compared on a relative scale with respect to EPC viscoelastic constants.

**Table 1:** Results from the Pressure-Dependent Extrusion Analysis for the Dynamic Viscosity, Young Modulus, and Flexure Modulus for All Liposome Suspensions Analyzed in This Work^a^

**Suspension**	**η (cPa)**	**η**_**relative**_	***E* (MPa)**	***k***_**c**_ **(× 10** ^**–19**^ **J)**	***K***_**c‑relative**_
EPC:Chol	0.96(13)	*1.00(10)*	55(5)	3.9(2)	*1.00(1)*
EPC	0.49(5)	*0.51(6)*	35(5)	2.5(1)	*0.63(2)*
DPPC	<630(60)	*<650(60)*	>110(5)	>7.8(2)	*>2.0(1)*

a Relative values of η and *k*_c_ are provided for comparison with defocusing microscopy results.

One notices from Table 1 that EPC and EPC:CHOL viscoelastic parameters present close values, allowing a comparison drawn relative to EPC:CHOL (used here as the reference system). The η value decreases by half from EPC:CHOL to EPC, while *k*_c_ is reduced by ∼40% in EPC. The abrupt difference from EPC:CHOL to DPPC is also clear in Table 1. Since DPPC extrusion took place only at large pressures, with a very slow flow rate, we believe that our extracted parameters represent an upper limit for η (large liposomes may partially block extrusion flow) and a lower limit for *k*_c_ (as-synthesized small liposomes pass the extrusion process and have an enhanced population in the DLS analysis).

In order to verify the validity of our results, the well-established technique of DM is used. Optical microscopy images of selected liposomes for EPC:CHOL and DPPC suspensions as observed in our DM setup are shown in Figure 6(a, b), respectively. One of the data sets used for the evaluation of the contrast time-dependent correlation of the liposome membranes for each suspension is depicted by open dots in Figure 6(c) for EPC:CHOL and in Figure 6(d) for DPPC. Fits using eq 5, represented by the solid red line in both cases, yield DM values for η and *k*_c_ listed in Table 2. One notices that absolute values extracted for η strongly differ from DM to the extrusion-viscosimetry (E-V) method developed here. Such a difference arises from the fact that MD directly measures the property of the liposome membrane while E-V results are based on the collective behavior of the liposome formulation. Nevertheless, MD finds a relative η value for DPPC approximately 10 times larger than the value retrieved for EPC:CHOL, showing that the difference between both formulations is considerably large (1 order of magnitude larger η at DPPC).

**Figure 6:**
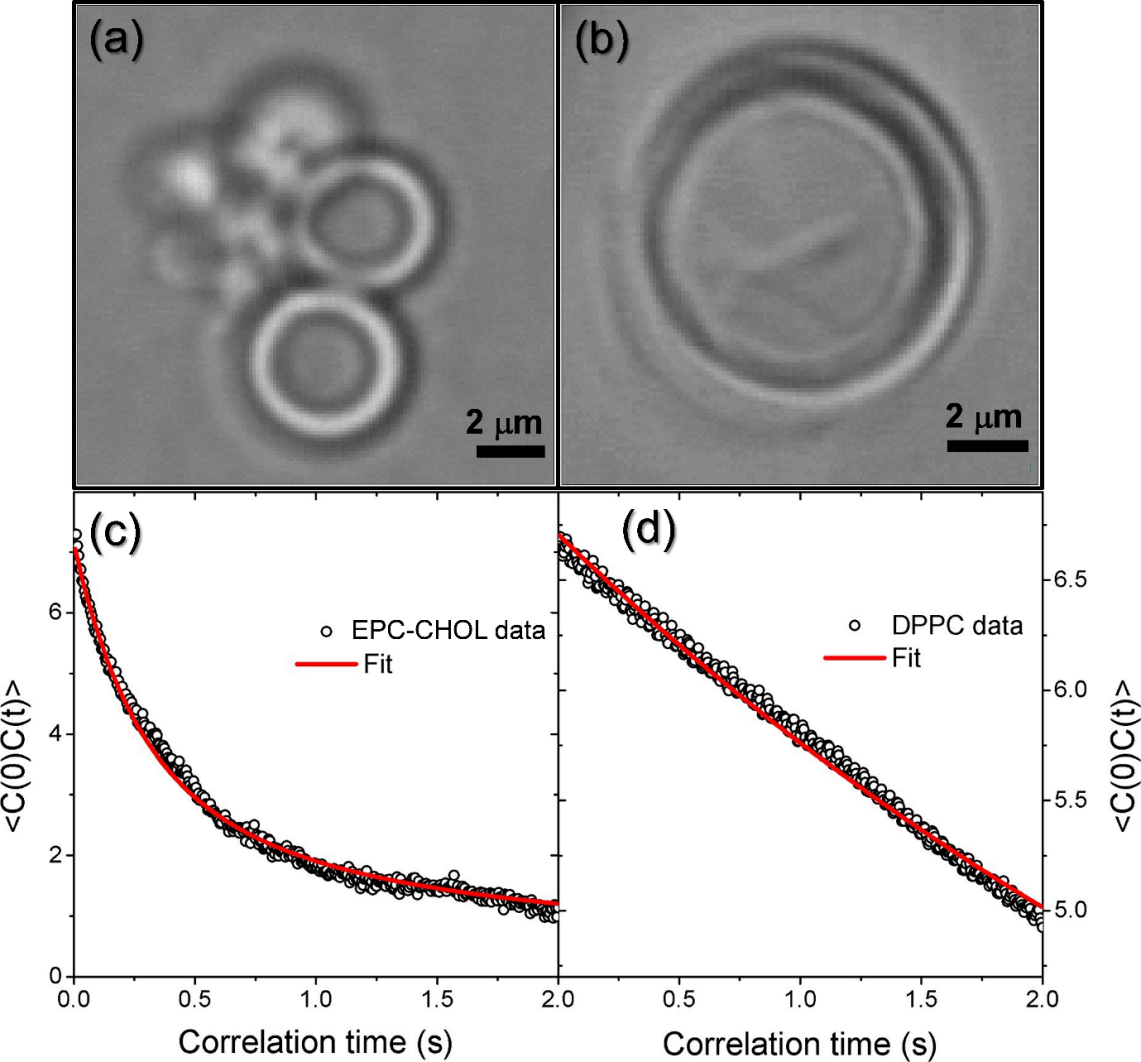
(a) Defocusing image of EPC:CHOL liposomes observed by optical inverted microscopy. (b) Defocusing image of a single LUV (large unilamellar vesicle) DPPC liposome observed by optical microscopy. (c) Temporal correlation decay and fit for one EPC:CHOL liposome. (d) Temporal correlation decay and fit for one DPPC liposome.

**Table 2:** Results from Defocusing Microscopy Analysis for the Dynamic Viscosity, Young Modulus, and Flexure Modulus for All Liposome Suspensions Analyzed in This Work

**Suspension**	**η (cPa)**	**η**_**relative**_	***E* (MPa)**	***k***_**c**_ **(× 10** ^**–19**^ **J)**	***K***_**e‑relative**_
EPC:Chol	260(3)	*1.00(1)*	145(5)	10.5(1)	*1.00(1)*
DPPC	2580(20)	*9.92(1)*	420(8)	29.8(1)	*2.84(5)*

For *k*_c_ values, the absolute numbers extracted for EPC:CHOL and DPPC from our E-V methodology are roughly a factor of 3 different from the MD results. However, when compared on a relative scale, MD results show a proportion of 2.84(5) considering the flexure modulus of both liposomes, while E-V relative results indicate a relative difference of 2.0(1). Such a finding is a clear indication that the analysis of the extrusion process by a combined viscosity and DLS pressure-stepwise procedure is a promising tool to compare liposome formulations, providing a consistent clue on changes in elasticity and viscosity behavior.

## VI Conclusions

We developed here a simplified methodology for the analysis of liposome extrusion using a set of tools that is commonly found in research laboratories. Our work is a valuable proof of concept that shows the possibility of simultaneous measurements of the Young modulus, viscosity, and flexure modulus if a series of controlled pressure experiments are carried out in the extrusion process. Using liposome formulations of broad interest for drug delivery, we have carried out stepwise pressure extrusion and DLS analysis. The first procedure, captured by a 120 fps video camera, allows for the retrieval of dynamic viscosity parameter η. DLS liposome fractions, discriminated in two size windows for each extrusion pressure series, were used to estimate the *k*_c_ parameter, using a simple approximation of multiplying by 100 the pressure of the inflection of the liposome population curve. Such an approach, which leads to a rough estimation of absolute values, is useful for obtaining fair relative flexure modulus values. If more precise procedures, based on the present work, require the analysis of a much narrower DLS size window (or exact peak positions in the DLS distribution), then it is crucial to notice that size deviations with respect to microscopy techniques must be considered.^53^

Further refinement of our method can be envisaged, and commercial tools may be developed from the principles discussed here. A proper design would possibly combine current extruder hardware with viscosimeter-inspired geometries for the output pipeline. An extrusion setup integrated with a finely constructed capillary system could be automated to carry out the pressure-series measurements shown here for distinct liposome solutions, eventually allowing the evaluation of absolute values for *E*, *k*_c_, and η (beyond relative values retrieved here). Our contribution is a valid starting point for future discussions and improvements in novel (and integrated) analysis techniques. We believe that our method can be a prequel to in vivo and in vitro tests whenever the biomechanical liposome conditions play a crucial role in drug delivery, considering systems in which suitable chemical parameters are investigated, but the drug release does not take place or happens before reaching the expected target due to encapsulation fragility or excessive toughness.

## Supplementary Material



## Notes

Most relevant DLS results along the extrusion pressure series, depicting the position of the two most intense DLS distribution peaks and width/percentage parameters for intensity and volume weighted distributions: intensity-weighted DLS distribution for the DPPC suspension (Table S1); volume-weighted DLS distribution for the DPPC suspension (Table S2); intensity-weighted DLS distribution for the EPC suspension (Table S3); volume-weighted DLS distribution for the EPC suspension (Table S4); intensity-weighted DLS distribution for the EPC:CHOL suspension (Table S5); and volume-weighted DLS distribution for the EPC:CHOL suspension (Table S6) (PDF)
