# Supplementary material for: Direct Retrieval of Biomechanical and Hydrodynamic Parameters for Drug Carrier Liposomes Using Conventional Extrusion Processes
Source: ACS Omega. 2026 Jan 20;11(4):6293–302. doi: 10.1021/acsomega.5c11079 (PMC12878738; doi:10.1021/acsomega.5c11079)
Supplement: Supplementary file 1 [file ao5c11079_si_001.pdf]

Supporting information to

Direct retrieval of biomechanical and hydrodynamic parameters for drug carrier liposomes using conventional extrusion processes

Maria Victoria Heiderick Machado[1], Maria Luiza Barbosa Pertence[1], Caroline Mari Ramos Oda[2], Jaqueline Aparecida Duarte [2], Ubirajara Agero[1], Elaine Amaral Leite[2], Angelo Malachias[1]*

[1] Departamento de Física, ICEx, Universidade Federal de Minas Gerais - UFMG, Av. Antonio Carlos, 6627, Belo Horizonte - MG, CEP 30123-970, Brazil

[2] Faculdade de Farmácia, Universidade Federal de Minas Gerais - UFMG, Av. Antonio Carlos, 6627, Belo Horizonte - MG, CEP 30123-970, Brazil

*Corresponding author: angelomalachias@gmail.com

In this supporting material we provide tables for the most relevant DLS results (generated by the Zetasizer-DLS equipment), depicting the position of the two most intense DLS distribution peaks and width/percentage parameters for intensity and volume weighted distributions. All tables shown here are averaged over the series of measurements for each formulation and each extrusion pressure. We recall that for our analysis one single extrusion step was performed for each pressure value in each suspension. The full measurement dataset consists of three measurements for each suspension (with 11 runs of 10s each). Considering all pressure measured, this corresponds to 27 intensity-weighted distributions and 27 volume-weighted distributions.

The tables presented below are not directly related to DLS analysis since our volume fraction evaluation provided in figures 4 and 5 for all investigated liposome suspensions are obtained after integration of the DLS volume distribution within large intervals (in semilog scale) of the DLS size distribution results. In our experiments the use of intensity-weighted or volume-weighted distributions for this integration leads to similar conclusions. The integration intervals, between 60nm and 300nm and between 400nm and 2500nm, are represented in figures 3(b – e) of the manuscript as vertical dashed lines.

Table S1 – DPPC suspension DLS results for intensity-weighted distributions

Pressure [kgf/cm^2]	Size (peak 1) [nm]	% Intensity	Std.Dev. [nm]	Size (peak 2) [nm]	% Intensity	Std. Dev. [nm]
8	410.9	97.2	93.8	24.7	2.7	14.7
9	660.2	99.6	154.6	5560.0	0.4	388.7
10	660.6	100	131.6	5460.3	0.0	580.0
11	614.6	98.5	177.6	5541.9	1.5	261.3
12	590.7	98.4	190.0	5490.8	1.6	198.2

Table S2 – DPPC suspension DLS results for volume-weighted distributions

Pressure [kgf/cm^2]	Size (peak 1) [nm]	% Volume	Std.Dev. [nm]	Size (peak 2) [nm]	% Volume	Std. Dev. [nm]
8	429.7	97.3	93.8	27.2	2.7	14.1

9	673.1	99.0	154.6	5553.2	0.6	193.3
10	661.4	100	131.6	5560.1	0.0	495.5
11	627.4	97.7	177.6	5552.7	2.3	394.0
12	603.9	97.6	190.0	5560.6	2.4	386.5

Table S3 – EPC suspension DLS results for intensity-weighted distributions

Pressure [kgf/cm^2]	Size (peak 1) [nm]	% Intensity	Std.Dev. [nm]	Size (peak 2) [nm]	% Intensity	Std. Dev. [nm]
2.5	785.0	45.8	429.7	2077.6	50.2	450.8
3.0	785.4	52.0	469.9	1947.8	46.7	492.4
3.5	953.1	57.3	632.1	1310.2	42.1	195.8
4.0	840.6	63.8	299.6	2141.5	34.0	544.0
4.5	652.0	69.6	388.2	2611.6	29.2	558.9
5.0	693.2	66.6	282.0	1782.6	31.0	336.0
5.5	730.8	78.0	381.7	3037.2	20.7	588.9
6.0	733.7	73.1	512.9	2237.3	26.8	750.4
6.5	578.6	70.3	238.8	2149.8	26.8	380.1
7.0	596.9	79.6	269.8	2053.1	18.6	742.1
8.0	557.6	82.7	349.3	3155.0	15.0	510.9

Table S4 – EPC suspension DLS results for volume-weighted distributions

Pressure [kgf/cm^2]	Size (peak 1) [nm]	% Volume	Std.Dev. [nm]	Size (peak 2) [nm]	% Volume	Std. Dev. [nm]
2.5	986.7	42.0	671.0	2126.6	56.5	352.7
3.0	949.5	52.8	556.7	3034.2	45.8	845.8
3.5	1020.1	59.5	810.1	2095.5	38.1	292.1
4.0	959.4	58.9	637.0	3402.8	39.8	669.2
4.5	772.0	65.2	440.5	2513.7	31.7	810.7
5.0	712.5	67.1	327.1	2467.7	26.9	859.6
5.5	783.6	78.3	436.2	3311.3	19.9	669.7
6.0	696.3	85.8	459.2	2703.2	14.2	630.9
6.5	657.7	69.1	273.2	2563.5	25.9	500.0
7.0	613.7	71.2	382.1	2796.6	25.0	874.4
8.0	544.4	79.2	405.1	2902.3	20.5	922.1

Table S5 – EPC:CHOL suspension DLS results for intensity-weighted distributions

Pressure [kgf/cm^2]	Size (peak 1) [nm]	% Intensity	Std.Dev. [nm]	Size (peak 2) [nm]	% Intensity	Std. Dev. [nm]
3.0	366.9	36.7	141.8	2740.3	62.3	413.9
3.5	870.3	52.4	410.1	2890.9	47.4	251.1
4.0	510.5	36.6	259.5	3319.3	63.4	382.0
4.5	420.7	65.2	152.8	4013.3	34.8	574.3
5.0	673.4	69.0	458.7	4276.0	31.0	726.6
5.5	557.0	62.8	286.7	3175.4	36.7	451.3
6.0	629.9	70.4	301.6	2983.3	28.9	621.9

6.5	652.7	78.7	309.1	3691.1	21.3	535.5
7.0	471.2	76.0	213.5	3058.3	24.0	819.3
7.5	465.8	86.0	186.1	3043.9	17.5	363.6
8.0	456.1	78.8	168.6	3983.0	21.2	505.9

Table S6 – EPC:CHOL suspension DLS results for volume-weighted distributions

Pressure [kgf/cm^2]	Size (peak 1) [nm]	% Volume	Std.Dev. [nm]	Size (peak 2) [nm]	% Volume	Std. Dev. [nm]
3.0	391.2	38.4	151.0	2476.8	55.5	394.9
3.5	802.5	47.2	408.4	2844.8	32.1	295.7
4.0	518.8	34.3	224.0	3396.3	65.7	663.6
4.5	455.2	62.9	163.4	3171.7	37.1	768.7
5.0	421.4	66.9	178.5	3285.6	33.1	754.7
5.5	550.2	69.6	214.6	3296.7	29.7	641.8
6.0	608.5	67.1	293.6	2621.7	31.6	548.8
6.5	548.3	74.4	268.6	3520.0	25.6	968.1
7.0	399.0	68.4	189.3	2427.5	31.1	461.5
7.5	496.0	82.4	181.9	2605.0	16.5	726.2
8.0	348.7	78.2	170.6	3781.5	21.8	521.9
